# Drawing up the public national Rational Pharmacotherapy Action Plan as part of social and health services reform in Finland: a bottom-up approach involving stakeholders

**DOI:** 10.1186/s12913-024-11068-y

**Published:** 2024-05-16

**Authors:** Heidi Tahvanainen, Liisa-Maria Voipio-Pulkki, Katri Hämeen-Anttila, Ulla Närhi, Taina Mäntyranta, Anna-Riia Holmström, Marja Airaksinen

**Affiliations:** 1 Drug Research Doctoral Programme, University of Helsinki, P.O. Box 56, 00014 Helsinki, Finland; 2 Ministry of Social Affairs and Health, Meritullinkatu 8, 00170 Helsinki, Finland; 3 School of Pharmacy, University of Eastern Finland, Yliopistonranta 1, 70210 Kuopio, Finland; 4 Health Emergency Preparedness and Response Authority (HERA), European Commission, Rue de La Loi 15, 1049 Brussels, Belgium; 5 Clinical Pharmacy Group, Division of Pharmacology and Pharmacotherapy, Faculty of Pharmacy, University of Helsinki, Viikinkaari 5 E, P.O. Box 56, 00014 Helsinki, Finland; 6 National Medicines Agency, Fimea, Mannerheimintie 166, 00300 Helsinki, Finland

**Keywords:** Rational pharmacotherapy, National medicines policy, Pharmaceutical services, Medication safety, Community pharmacy, Integration of services, Social and health services reform, People-centeredness, Partnership, Evidence-informed policy

## Abstract

**Background:**

Ensuring equal access to medicines and their appropriate and safe use at reasonable costs are core functions of health systems. Despite that, few descriptions of national medicines policies' development processes and implementation strategies have been published. This study aimed to describe the government program-based development of the Rational Pharmacotherapy Action Plan in Finland as a part of the undergoing major health and social service system reform, also covering the implementation of rational pharmacotherapy in the reformed system and processes.

**Methods:**

The data of this qualitative study consisted of public reports and Steering Group meeting memos related to the development of the national Rational Pharmacotherapy Action Plan that the Ministry of Social Affairs and Health coordinated. Qualitative content analysis applying systems theory and the conceptual framework of integrated services as theoretical frameworks was used as an analysis method.

**Results:**

The national Rational Pharmacotherapy Action Plan covering 2018–2022 was created in a bottom-up development process involving a wide range of stakeholders. Rational pharmacotherapy was redefined by adding equality as the fifth pillar to complement the previously defined pillars of being effective, safe, high-quality, and cost-effective. The Action Plan formed a normative framework for long-term development, with a vision and principles focusing on people-centeredness, better coordination and management of the medication use processes, the continuity of treatment paths and the flow of patient and medicines information through partnerships, and evidence-informed policies and practices.

**Conclusion:**

Through intensive stakeholder participation, the bottom-up approach created a national vision and principles of rational pharmacotherapy along with strong commitment to implementing the goals and measures. The concern lies in ensuring the continuity of the Action Plan implementation and achieving a balanced long-term development aligned with the integrated and reformed national social and health services system. The development of the pharmaceutical system has several national and EU-level dependencies requiring political long-term commitment. While the Action Plan differs from the national medicines policy, it forms a good basis for long-term development covering important parts of medicine policy at the micro, meso, and macro levels of the service system.

**Supplementary Information:**

The online version contains supplementary material available at 10.1186/s12913-024-11068-y.

## Background

National health systems may have several goals, but the ultimate is improving population health and well-being [1]. According to the World Health Organization (WHO), an effective health service system should meet these goals by providing equal access to affordable and high-quality services, including care and healing services, health promotion, prevention, and rehabilitation for the entire population [1, 2]. Access to medicines is essential to a well-functioning health system and is necessary to achieve public health goals [1, 3]. When successful, pharmacotherapy can save lives, maintain, or improve functional capacity, and mitigate or even prevent diseases or their symptoms, thus improving quality of life. The demand for and spending on pharmaceuticals are expected to grow due to aging populations, rising income levels, increasing costs of developing new technologies, and increased patient expectations [4]. Consequently, pharmaceuticals constitute a substantial portion of healthcare spending in Europe and globally. In Finland, pharmaceuticals accounted for 14–15% of the total social and health services costs in 2021 [5].

Many countries have experienced challenges in meeting all health service demands. Health inequalities, limited availability and access to services, safety, and productivity are the main shared concerns [6–8]. In the last decade, a major social and health services reform has been prepared in Finland, aiming to improve the coordination, integration, and equality of access to services while balancing continuously growing health and social services costs [9, 10]. In many countries, including Finland, the government or another third party pays a significant part of pharmaceutical costs. Therefore, effective means are required to monitor and guide the safe and appropriate use of medicines and their cost-effectiveness. Pharmacotherapy can be considered inappropriate if it does not meet the conditions and core components of rationality defined by the WHO [11, 12]. According to the WHO, rational use of medicines occurs when patients receive medications appropriate to their clinical needs, in doses that meet their own individual requirements, for an adequate period, and at the lowest cost to them and their community [11]. Due to the complex interrelationships of pharmaceutical and health system governance, countries have been recommended to find ways to harmonize better and align their pharmaceutical policy activities with national health policies and systems [1, 13].

As part of a national policymaking, the need for rational pharmacotherapy programs has been recognized in several countries [4, 14, 15]. Few detailed descriptions of national medicines policies (NMP), development processes, and implementation strategies are available. However, such descriptions would benefit other countries in compiling their own policies [14, 16] and developing international policy recommendations [3, 4]. According to WHO, a NMP serves as both a commitment to a defined objective and a strategic roadmap for actionable steps [13]. This national comprehensive framework articulates and prioritizes the government's medium- to long-term goals for the pharmaceutical sector and use of medicines, outlining key strategies to achieve these objectives. For example, NMP has guided development activities in Australia since 2000 [16]. The overarching goal of the Australian’s NMP has been to optimize health outcomes through a collaborative partnership with key stakeholders. A similar target has been set for the NMP implementation in New Zealand [17]. In 2022, the updated Australian’s NMP has emerged as a coordinating framework that sets out a vision, common aim and intended outcomes, for all partners to work towards quality use of medicines and medicines safety by focusing on the current and future health needs of people in Australia [18].

In Finland, the development of the pharmaceutical system has been guided by the NMP since 2003 [19]. The NMP was originally drafted to evaluate the development needs of the pharmaceutical system in a situation where medicine legislation had not been comprehensively assessed for an extended period. Moreover, Finland had joined the European Union (EU) in 1995, resulting in the harmonization of national legislation with EU regulations. The purpose of the NMP was to bring predictability in the operating environment to several stakeholders in the medicines sector. In the 2011 NMP update, Finland implemented the WHO's recommendation to commit various key stakeholders to NMP goals by involving them in an open, systematic consultation process when preparing the NMP [13, 20]. By doing so, Finland’s goal has been to develop the pharmaceutical sector and service system aligned with the health policy goals to meet the needs of the social and health services [21]. However, rapidly rising pharmaceutical costs, an aging population, and medication safety risks, especially among older adults, a fragmented operating system and culture, as well as pressures to promote the digitalization of healthcare formed complex challenges to be solved as part of the social and health services reform [22–24]. Therefore, the Ministry of Social Affairs and Health (MSAH) initiated in 2016 the preparation of the targeted Action Plan promoting rational pharmacotherapy based on the government mandate [25].

The aims of this study were to 1) describe the collaborative and bottom-up development process of the national long-term Rational Pharmacotherapy Action Plan during 2016–2017 and the key process outcomes, and 2) to analyze the content of the Action Plan in the conceptual framework of integrated care.

## Method

### Context of the study

Finland has a population of 5.6 million, of which 1.7 million (30%) live in the metropolitan area of Helsinki [26]. Life expectancy at birth is one of the highest in the world, with 84.5 years for women and 79.2 years for men in 2021 [27]. GDP per capita was about 47,991 euros in 2022 [26]. Social security and access to health services are considered universal residents’ rights according to the Constitution of Finland [28].

Finland has a public healthcare system, complemented by private and occupational healthcare services [9, 29]. The ongoing reform restructures the organization of public healthcare and social welfare systems, and rescue systems [10]. The aim of the new legislation is to ensure equal, interoperable, and cost-effective healthcare and social welfare services throughout the country. Additionally, the objective is to strengthen basic-level services and ensure better support for those who require a variety of social and healthcare services. In Finland care for the older people and substance abuse services are part of social welfare services unlike some other countries. Thus, integration and coordination of healthcare and social welfare services are essential and pharmacotherapy is part of this integration. In January 2023, the responsibilities of primary and secondary care were transferred from municipalities and hospital districts to the well-being services counties (*n* = 21) [10, 29]. In addition, the City of Helsinki (the largest city) became responsible for services in its own area. The District of Helsinki and Uusimaa is responsible for specialized healthcare in the metropolitan area. Finland is divided into five collaborative areas for tertiary care, each with a university hospital [9]. The well-being services counties may either provide services, act in cooperation, or purchase services from private service providers [10]. The well-being services counties receive state funding according to the criteria set in legislation and supplement their finances with service fees, highest amounts of which are set in the legislation. They do not have the right to collect taxes to cover social and healthcare costs [10].

Pharmacotherapies conducted during hospital care are included in patient service fees, financed by the well-being service counties. In contrast, medication use in outpatient care is mainly covered by the public social insurance funded jointly by the government and the insured individuals (covering equally all permanent residents in Finland) [9]. Medicines for outpatient care are dispensed from community pharmacies and are partially or fully reimbursed by public social insurance, covering equally the entire population [9, 30]. The reimbursement scheme for pharmacotherapy is disease-based and offers relatively high deductibles for long-term medicine users of chronic diseases [9].

In accordance with health service legislation in Finland, all operations are grounded in evidence-based practices [31]. The responsibilities related to informing decision-making through Health Technology Assessment (HTA) are decentralized among various organizations. In particular, the assessment and decision-making for outpatient care medicines fall under the jurisdiction of the Pharmaceutical Pricing Board [32], while new medicines for inpatient care are evaluated by the Finnish Medicines Agency (Fimea). Recommendations to introduce medicines in inpatient care are provided by the Council for Choices in Health Care [33]. Also, indication extensions of medicines are subject to HTA, coordinated by the Finnish Coordinating Center for Health Technology Assessment, with assessments conducted on a hospital level [34]. Furthermore, the decision-making has been informed by research conducted by several stakeholders: universities, state research institutes, agencies and institutions of various administrative branches, and advocacy organizations.

Since 2014, innovation activities in the medicines and health sector have been guided by the Health Sector Growth Strategy for Research and Innovation, along with its subsequent update, the Roadmap [35], prepared in cooperation with the Government and research and innovation funders and organizations in the health sector. The advancement of pharmaceutical innovations is crucial for treatment development and addressing unmet medical needs. Investing in innovation activities not only facilitates sector growth but also has the potential to boost health sector exports, a significant aspect of research, innovation, and industrial policy, particularly in Finland, where a considerable portion of medicines is imported [35]. The overarching focus of health sector policy and innovations in Finland has primarily centered on enhancing the ecosystem for personalized medicine [36].

### Medicine use in outpatient care

In Finland, community pharmacies have remained the sole source of prescription and non-prescription medicines to outpatients with the exemption of nicotine replacement therapies released to open sale in 2006 [37]. Pharmacy operations are subject to licensing; a pharmacy owner must have at least a MSc (Pharm) degree, sufficient experience in pharmacy operations, prerequisites for running a pharmacy business and not been declared bankrupt or legally incompetent [38]. Community pharmacies are legally obligated to maintain an adequate supply of medicines that address the population’s needs within their operational area. Additionally, they must maintain adequate pharmaceutical personnel to fulfill certain duties, e.g., medication dispensing and counseling for both over-the-counter (OTC) and prescription medicines.

The retail prices of all medicines and the wholesale prices of medicines included in the reimbursement scheme are regulated [38–40]. The objective of regulating medicines price is to establish fair and equitable prices that benefit both medicine users and society at large. Furthermore, this regulatory framework sustains competition within the market for interchangeable medicines and shifts the focus of competition among community pharmacies primarily towards quality of customer service rather than pricing strategies. Price regulation ensures that community pharmacies of varying sizes can procure wholesale medicines at uniform rates, thereby enhancing vertical transparency in the distribution chain. Generic substitution was implemented in 2003, a reference price system in 2009, and the automatic substitution of biologics will be gradually implemented in the beginning of 2024 in outpatient care to enhance price competition and reduce medicines costs [41, 42]. Since 2022, price regulation has made it possible to give discounts on the retail prices of OTC medicines [43]. Finland largely depends on imported medicines since there is no strong domestic pharmaceutical industry [44].

Since 2017, all outpatient prescriptions have been issued and dispensed electronically via a national electronic health record system, Kanta, maintained by the National Social Insurance Institution Kela [45]. Kanta is an entity of national patient information depository and data management services used by citizens, social and health service providers, and pharmacies [45]. It allows centralized use, storage, and maintenance of electronic patient data, and data exchange for cross-border purposes [46, 47]. Citizens have adopted the use of Kanta services well and can browse their own medical records and prescriptions and, e.g., order repeat prescriptions through the online service [47]. The development and interoperability of national information management services and information management systems in social services and healthcare are guided and coordinated nationally [48].

### Origins of the Rational Pharmacotherapy Action Plan

The prevention and mitigation of inequality, improving the availability, access, and continuity of care, and cost growth management have become increasingly important guiding principles in Finnish health policymaking during recent decades, reflected also in the NMP [10, 20, 29]. According to the NMP published in 2011, rational pharmacotherapy and good medication safety enhance people's well-being, improve public health, and decrease healthcare expenditures [20]. However, achieving the NMP goals had become more challenging, especially due to rapidly increasing medicines costs [23], the unequal and uncontrolled introduction of new pharmaceutical products [49] and medication safety risks, especially in the vulnerable population groups such as older adults [22, 24]. Thus, the government program for 2015–2019 mandated the MSAH to establish a Rational Pharmacotherapy Action Plan (RPAP) [25]. The goals set for the Action Plan by the Government were to improve comprehensive patient care, improve people's functional capacity, and create conditions for cost-effective pharmacotherapy from the perspectives of the patient and society [50]. At the same time as the Action Plan was drawn up, social and health services reform was being prepared, which also covered the development of the pharmaceutical system aligned with the renewed social and health service system. It is to be noted that during the development of the Action Plan, no policy guidelines or legislation for restructuring the pharmaceutical system were available.

### Theoretical framework of this study

System theory, also known as systems thinking, provides a holistic approach to understanding and evaluating complex phenomena, e.g., within social and health systems [1, 51]. According to the system theory, all interventions tend to generate effects at the system level [51]. Different systems interact with each other but retain their autonomy due to the structures and processes of the system [1, 3, 51].

Integrated health systems are considered as a solution to maintaining accessibility, quality, and continuity of services [52–54]. System integration requires a tailor-made combination of structures, processes, and techniques to meet the service needs of people and population [54]. For example, functional integration includes mechanisms that establish connections between services through funding, information, and management. Normative integration consists of informal coordination mechanisms of mission, vision, values, and culture, which promote integration, if these have been successfully shared as a common set of goals at all levels of the system [54]. Different functions and processes at the system’s levels: the macro (system), the meso (organizational and professional) and the micro (clinical), complement each other to achieve integration goals [54].

In this study, the system theory [51] guided interpretations of the interaction between different parts of the pharmaceutical and health service systems. At the same time, the conceptual framework of integrated care [54] was applied to analyzing integration of functions between different actors in the medication use process at macro, meso and micro levels.

### Study design and methods

This study is based on qualitative content analysis of the final report of the Rational Pharmacotherapy Action Plan (RPAP) [50] and the meeting memos of the Steering Group responsible for the development of the RPAP under the leadership of MSAH [55–57]. All reports published during the development process of RPAP were utilized in the analysis to verify the interpretations of the content analysis (Documents no. 3–13, Table s1 in Additional File 1). The interpretation was also influenced by approved legislation of the national social and health services reform [58], the national programs that preceded the RPAP such as NMP 2020 [20], National recommendation for multidisciplinary cooperation in optimizing pharmacotherapy in older adults [24], Medicines information strategy 2020 [59] and Government resolution of patient and client safety strategy 2017–2021 [60]. In addition, recent developments under the initiative of the European Health Union [61] influenced the interpretation, e.g., the implementation of the European Pharmaceutical Strategy since 2020 [62], the Regulation on Health Technology Assessment [63], the proposal of the European Health Data Space including data and information development for cross-border healthcare and prescription development [64], and changes in the responsibilities and processes of the various EU Agencies [65]. Above mentioned reports describing the development of the operating environment were reviewed alongside the analysis to understand connections and dependencies.

### A qualitative synthesis of data

The first part of the study was an inductive content analysis of the Steering Group meeting memos and the final report of the RPAP (Documents no. 1–2, Table s1 in Additional File 1). The analysis focused on describing the development process of the Action Plan and the outcomes of the development process. The second part was a deductive content analysis of the key contents of the Action Plan based on the final report of the RPAP and Steering Group meeting memos. The system theory guided the analysis and thinking concerning how the structures and processes of the pharmaceutical system interacts as part of the social and health service system at the micro, meso and macro levels [51]. The conceptual framework of integrated care guided the analysis related to the medication use process [54].

First, the main themes of the final RPAP report and Steering Group memos, as well as the concept of rational pharmacotherapy were reviewed and refined to draw connections between NMP and broader social and health policies in the context of long-term development [10, 20, 21, 58, 59, 66]. Then, the prioritized actions and principles to promote rational pharmacotherapy were identified. The prioritized actions were classified into the following functional integration categories of integration framework: funding (F), information (I), and management (M), depending on how the implementation of the prioritized action can be promoted. Working Group (WG) and expert reports published during the RPAP development process were utilized to verify the interpretations (Documents no. 3–13, Table s1 in Additional File 1, Table s3 in Additional File 6). The classified actions were cross-tabulated with the key principles outlined by the Steering Group and presented in the RPAP and the different interacting levels of the system, i.e., micro, meso, and macro levels [51, 54]. The long-term development visions for the system’s micro, meso, and macro levels were condensed based on the abstraction of prioritized actions and principles [51, 54]. The EU level was considered at the system levels as the development of EU policy strongly influences the national development priorities of each Member State [62–65].

**Fig. 1:**
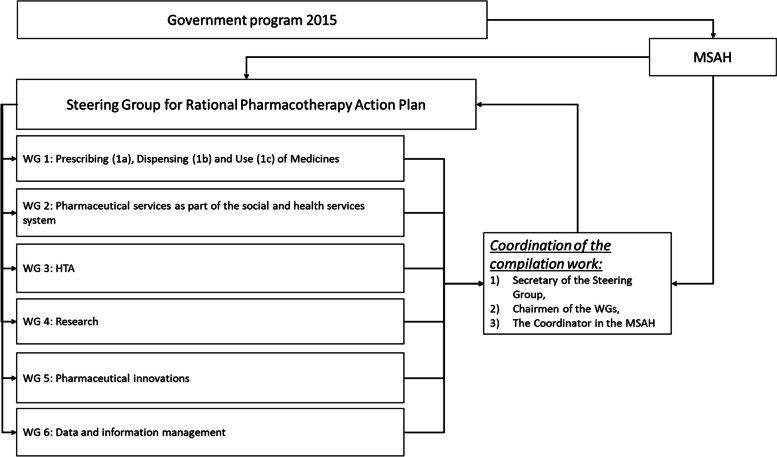
Organization of the Rational Pharmacotherapy Action Plan development. MSAH = Ministry of Social Affairs and Health, HTA = Health Technology Assessment, WG = Working Group

One researcher (HT) was responsible for the analysis, and other co-authors who were involved in the development process of RPAP (MA, KHA, UN, LMVP, TM) verified the validity of the analysis [57]. Author (UN), working in the MSAH at that time, launched the development project of the RPAP together with the chairman of the Steering Group, author (LMVP), who served at that time as director general in the MSAH. Author (HT), working in the MSAH at that time, coordinated the development of the RPAP and the compilation of the final report during 2017–2018. Author (KHA), working at that time in the Fimea, served as secretary of the Steering Group during 2016–2018 and was responsible for preparing the final report. Author (MA), professor at the Helsinki University, represented researchers to bring scientific evidence to the development process, served as chairman of the Research Working Group (WG) and member of the Steering Group, and author (TM), senior ministerial adviser in MSAH, brought information about the progress of social and health services reform to the development process of the RPAP, and served as chairman of the WG1 Prescribing, Dispensing and Use of medicines (Fig. 1).

## Research ethics

Good scientific practices were followed throughout the research process [67]. Only reports available from open sources were used. The meeting memos were made available through a formal information request to the MSAH.

## Results

### Description of the development process of the Rational Pharmacotherapy Action Plan

**Fig. 2:**
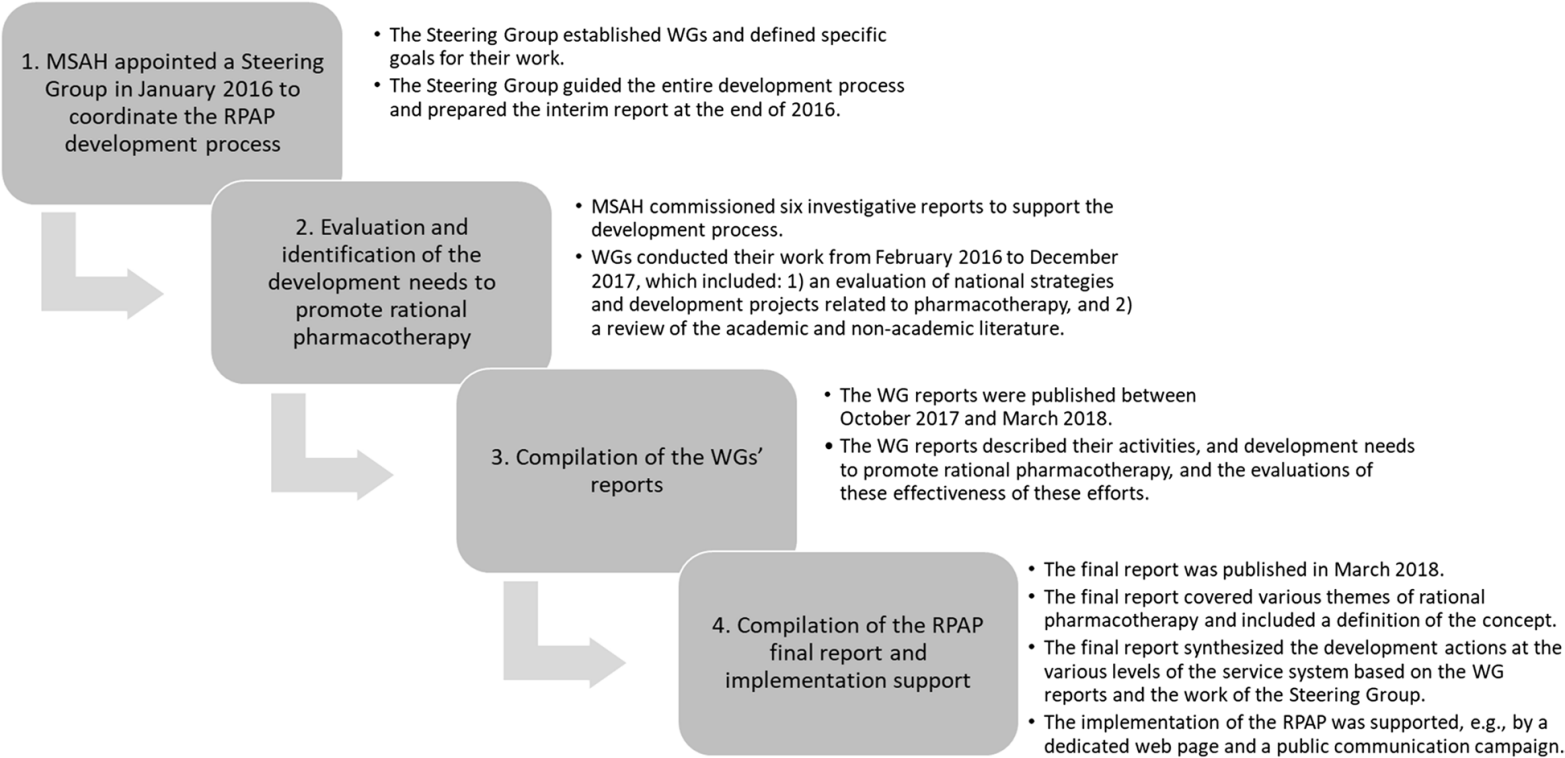
Progression of the Rational Pharmacotherapy Action Plan development. MSAH = Ministry of Social Affairs and Health, RPAP = Rational Pharmacotherapy Action Plan, WG = Working Group

In January 2016, MSAH appointed a Steering Group to coordinate the RPAP development process, which consisted of four phases (Fig. 2). The aim was to create a long-term RPAP for national implementation using a bottom-up approach in cooperation with a wide range of stakeholders involved in the planning, policymaking, and implementation of social and health services.

In the first phase, the Steering Group identified Finland’s current state and major challenges in rational pharmacotherapy. It established the following six WGs accordingly: WG1 Prescribing, dispensing and use of medicines, WG2 Pharmaceutical services as part of the social and health services system, WG3 Health Technology Assessment (HTA), WG4 Research, focusing on the ongoing and needed research in rational pharmacotherapy, WG5 Pharmaceutical innovations, and WG6 Data and information management (Fig. 1). The development needs of data and information management (WG 6) were derived from the work of each of the five WGs. The Steering Group set concrete goals for the WGs and guided the work by analyzing the current situation in regular follow-up meetings. An interim report was prepared by the Steering Group (Fig. 2).

A total of 40 stakeholder organizations and approximately 100 representatives participated in the development process. A diverse group of health and pharmaceutical experts and managers, authorities, and researchers were identified and invited by the MSAH to participate in the development work and WGs over two years. The following national umbrella organizations and stakeholders were involved in the Steering Group and WGs: public administrations, the major public third-party payor and government funding bodies, HTA bodies, civil servants working on the national preparation of the social and health care reform, organizations responsible for primary and secondary care (hospital districts), professional organizations and scientific societies, organizations representing pharmaceutical industry and community pharmacies, physicians, nurses, universities, hospital pharmacies, patient organizations and associations and representatives of the pensioners.

The work was based on a bottom-up activity that aimed to consider the interests of the various stakeholders in the field, even those with conflicting ones. The interaction with those involved in preparing the national social and health services reform progressing in parallel was regular and intensive. Similarly, the utilization of research results was a consistent practice. WG 4 mapped the ongoing research promoting rational pharmacotherapy, the researchers and research groups conducting the research, their view on future research needs and the need to improve research prerequisites and cooperation. The WGs used workshops, interactive seminars, invited experts for hearings, and extensive comment rounds of the draft documents in preparation. The overarching objective was to increase participation and commitment.

In the second phase, the WGs defined development measures based on assessments of the current state of rational pharmacotherapy, including administrative processes, strategies on patient safety [60] and medicines information [59], evidence of recent national development projects [24] and academic and non-academic literature (Fig. 2). The activities of the WG 4 supported the work of other WGs and enabled intensive utilization of research results. In addition, the following investigation reports were commissioned by the MSAH to inform the RPAP development: 1) The effects of EU legislation on national pharmaceutical system (not published), 2) The effects of social and health services reform on hospital pharmacy operations, 3) Steering for rational prescribing in selected countries, 4) Description of the regional organization and tasks of pharmaceutical working groups in Sweden, and 5) The patient-specific optimization of pharmacotherapies and the possibilities of information systems to support different phases of the medication management process (Reports no. 9–13, Table s1 in Additional File 1). The regular interaction with the preparation of the national social and health services reform progressing in parallel guided the work of WGs 1, 2, and 3.

In the third phase, each WG compiled a separate report on action proposals to promote rational pharmacotherapy (Fig. 2). The development needs for data and information management was also compiled in a separate report based on the findings of each WG. A total of 13 reports were compiled to guide the development and promote rational pharmacotherapy at different levels of the service system (Table s1 in Additional File 1). In the reports, comprehensive information was collected on national topics of rational pharmacotherapy, such as HTA operations and the utilization of its results in decision-making, as well as operating models and information needs of medicine users and different professionals in the different phases of the medication use process (e.g., the practices of collaborative medication review and optimization of pharmacotherapy, as well as the monitoring and documentation of the outcomes, as well as the division of work between different professionals). The existing national research evidence was used extensively to inform the work of different WGs, especially concerning the operation models and practices of safe and appropriate pharmacotherapy. Examples of rational pharmacotherapy steering methods were compiled from the sources described in the reports.

In the fourth phase, the secretariat of the Steering Group, the Chairpersons of each WG, and the coordinator of the RPAP program compiled the final report of the RPAP (Fig. 2). The writing process was contributed by constant discussion and reflection between the secretariat of the Steering Group (KHA), coordinator of the RPAP (HT), and the Chairpersons of each WG to grasp multiple perspectives and views of the stakeholders participating in the development work. One part of this phase was to redefine the concept of rational pharmacotherapy and legitimize it on the national level.

### Outcomes and core contents of the Rational Pharmacotherapy Action Plan development process

The final report [50] of the RPAP was published in March 2018, and it was planned to cover a period until the end of the next government term, 2022. The main content of the Action Plan was visualized as a house, where the structures and governance of the service system and evidence form the foundation for the people-centered services and partnerships in the medication use process and management of the medication regimen (Additional File 2). In the RPAP, the roles of different professionals and medicine users in the medication use process were defined and described for the first time in a national document to illustrate the complexity and vulnerability of the flow of information in the medication use process in outpatient care (Additional File 3). During the preparation of the RPAP it was found that professionals need support in optimizing and managing pharmacotherapy in dialogue with the medicine users. Therefore, a set of questions was created to support the discussion of rational pharmacotherapy with medicine users on a partnership basis (Additional File 4).

**Fig. 3:**
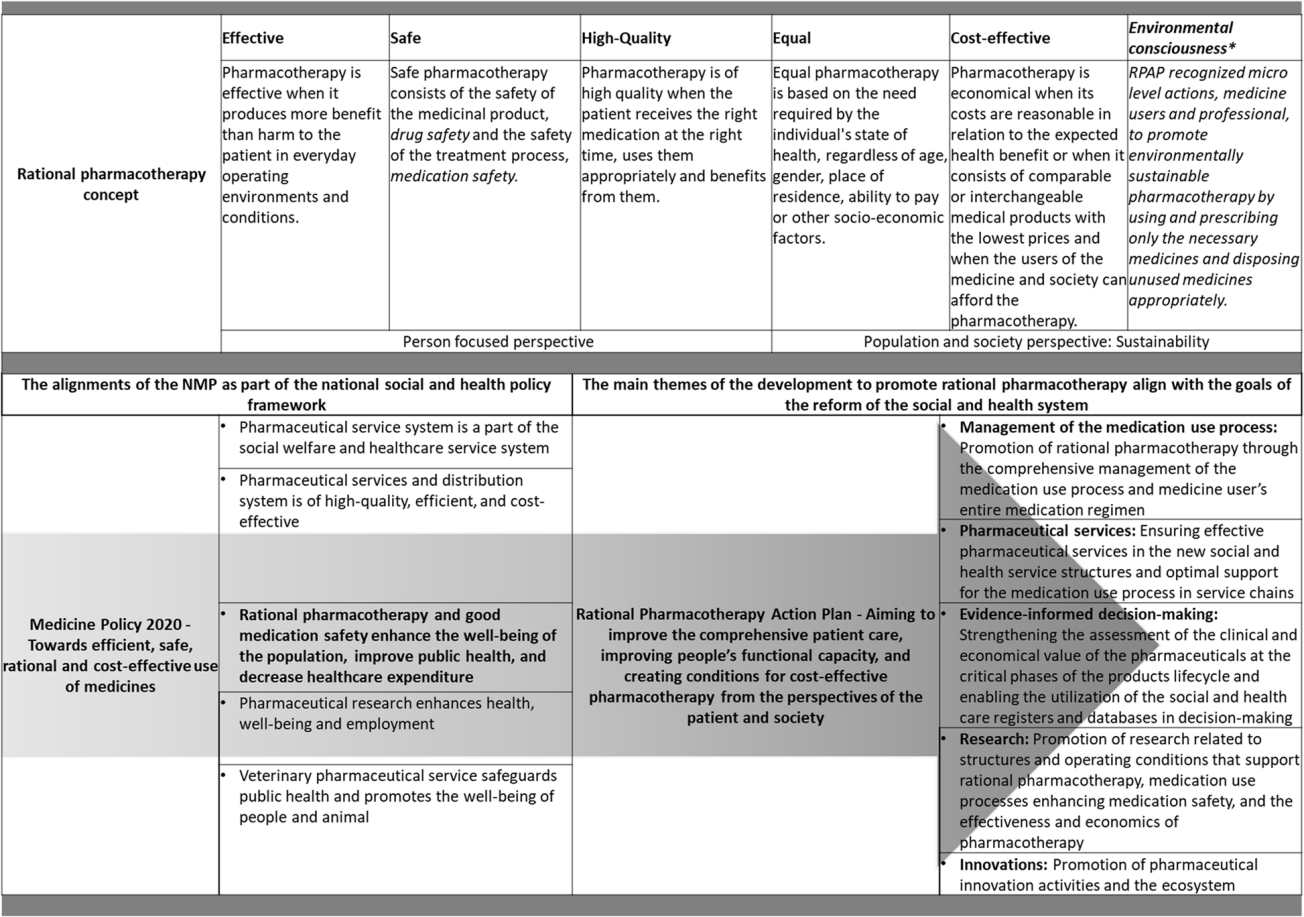
The rational pharmacotherapy concept and long-term policy themes [20, 50]. * Italic used to mark dimension not included in the definition of rational pharmacotherapy in RPAP. NMP = National Medicines Policy, RPAP = Rational Pharmacotherapy Action Plan

Based on the RPAP final report [50] and national policy documents [10, 20, 21, 59], the recognized main themes of long-term medicines policy development to promote rational pharmacotherapy were the management of the medication use process, pharmaceutical services, evidence-informed decision-making, research, and innovations (Fig. 3). In the Action Plan, rational pharmacotherapy was redefined through five dimensions (Fig. 3). Equality was added to rational pharmacotherapy to complement the four previous elements: effective, safe, high-quality, and cost-effective. Based on the Steering Group memos, the goals of effectiveness and equality were especially emphasized. The dimension of environmental awareness was not included in the RPAP definition of rational pharmacotherapy although it was noticed by the measures aiming to reduce waste in the medication use process.

**Fig. 4:**
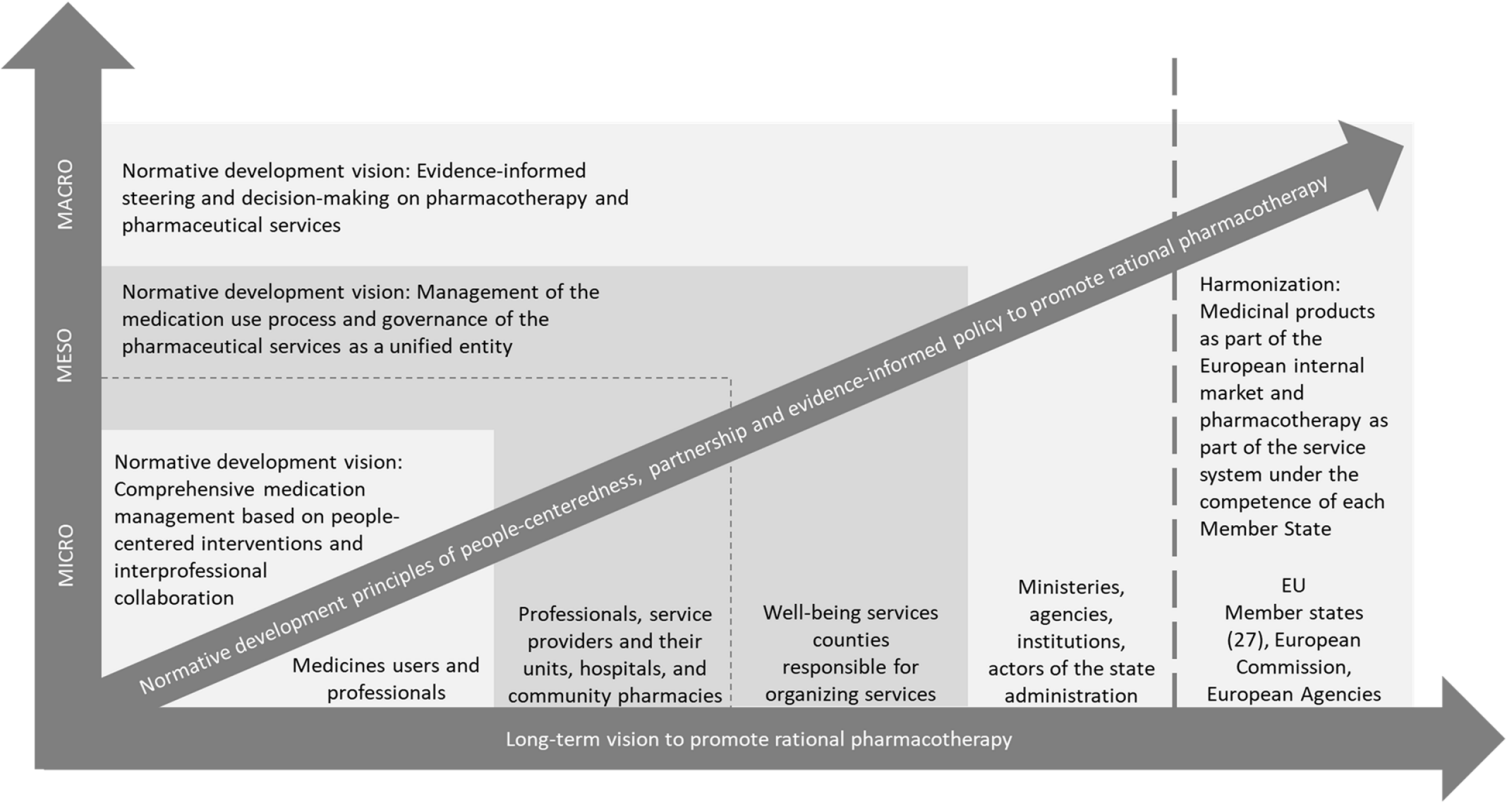
Long-term normative vision and principles to promote rational pharmacotherapy under the conceptual framework of integrated care [54]. EU = European Union

Based on the Steering Group memos, RPAP aimed to respond to identified challenges in the coordination, appropriateness, and continuity of pharmacotherapy by emphasizing people-centeredness, partnership, equality, and improved management of the medication use process and patient-specific medication regimen. The crystallized long-term normative development visions based on the RPAP final report [50] for the different levels of the service system were 1) micro: comprehensive medication management based on people-centered interventions and interprofessional collaboration, 2) meso: management of the medication use process and governance of the pharmaceutical services as a unified entity, and 3) macro: evidence-informed steering and decision-making on pharmacotherapy and pharmaceutical services (Fig. 4).

Based on the RPAP final report [50] and the WG reports (reports no. 3–13, Table s1 in Additional File 1), the visions and principles of the Action Plan aimed at improving the coordination and management of the medication use, the continuity of treatment paths, the flow of patient and medicines information and the effective use of knowledge resources (Table s2 in Additional File 5). At the micro level, this requires a review of the tasks and responsibilities of medication users and different professionals in the people-centered, partnership-based medication use process. The development on the micro level emphasizes ensuring continuity of pharmacotherapy, improving the management and monitoring of the medication regimen, and enhancing the clinical decision-making based on comprehensive patient and medicines information as part of the development of treatment paths and information systems.

For the service organizers, currently the well-being service counties, the Action Plan set several goals for governance, coordinating the medication use process, and creating conditions for clinical work that utilizes the expertise of different professionals. Coordinated and integrated service production, as well as research and development activities utilizing information of national and local registers aim to support the continuous development of clinical and medication safety practice and targeting of pharmacotherapy and clinical pharmacy services to those who benefit the most (Table s2 in Additional File 5). According to the Action Plan well-being services counties should establish effective means and structures for service production steering, control, and continuity of care also considering community pharmacy services and resources to ensure rational use of medicines and the implementation of national guidance. Evidence-informed steering and decision-making require successful development of regional information systems, utilization of information and interoperability with national data repositories and information management services. The simultaneity and mutual understanding of meso and macro level development was noteworthy in the Action Plan.

The need to improve governance and steering was also emphasized at the macro level, where several authorities related to pharmacotherapy operate. Also, the need for developing the division of work and the roles of different authorities was identified in the Action Plan. At the macro level, it was noted that the development of the harmonization and exchange of information and the overall administration of the pharmaceutical system extend to the EU level. However, considering the competence of each Member State (Fig. 4, Table s2 in Additional File 5). It was found that implementing the prioritized actions of the RPAP would require purposeful development at different levels of the system. This entails further development focusing on enhancing the functional integration of social and health services and the pharmaceutical system, utilizing funding, information, and management resources effectively.

### Implementation

Based on the Steering Group memos, the implementation of the Action Plan was aimed to support in various ways to create awareness of the redefinition of the rational pharmacotherapy and the set development goals. This aimed to get the development started both by different stakeholders and at different levels of the system. MSAH allocated quite a lot of time and resources for establishing an awareness campaign held in two phases, first targeted to social and health professionals and then to the public. The campaign messages and materials were designed in cooperation with the RPAP Steering Group and stakeholders. Implementation was carried out jointly with Fimea. In addition, MSAH launched a dedicated rational pharmacotherapy website rich in content in early 2018 [68].

## Discussion

Drafting the RPAP was an intensive, time-consuming, but fruitful national process in Finland. It enabled a comprehensive review of the current pharmacotherapy practices and, based on that, the creation of the long-term vision of rational pharmacotherapy as part of the planned social and health services reform. Although final legal decisions on the future of the social and health services system and the structures of the pharmaceutical system integrated into it were missing during the planning phase of the RPAP, it was possible to define guiding visions and principles for the promotion of rational pharmacotherapy in the micro, meso and macro levels of the service system (Fig. 4).

A variety of authorities and stakeholders participated in the development of the RPAP. This bottom-up and co-creation process was chosen because of the good experience gained from preparing the NMP 2020 [20]. This approach has been recommended by the WHO [13], and later used to support EU pharmaceutical decision-making [62]. The chosen development process of the Action Plan has proved to be important for the stakeholder commitment and enabled consensus of the visions and principles that still carry the development. This reinforces the earlier finding that the policy process is just as important as the policy document since the process must create a mechanism to bring different views together and achieve a sense of shared ownership of the outcome [14]. For example, the rational pharmacotherapy website continues to be actively managed by MSAH. Plenty of practical tools developed to promote rational pharmacotherapy in everyday clinical practice have been identified and shared through coordination with Fimea [69] and national medication safety development network [70]. In addition, Fimea has integrated the RPAP communication campaign into Pharmacotherapy Day’s annual campaign week, which is now organized in collaboration with different stakeholders [71].

During the development of the Action Plan, there was a thorough discussion about the definition of rational pharmacotherapy which led to the extension of the concept towards equity. That reflected the national principal value base of equal rights, which had become threatened by rapidly increasing healthcare and pharmacotherapy costs. Consequently, in the 2000s, Finnish pharmaceutical policy and decision-making on pharmaceuticals revolved around how to curb the ever-increasing costs. To control costs, changes have been made, e.g., to the public medicines reimbursement system and pricing, which have led to relatively high deductibles for long-term medicine users [9] and may have led to a decline in the adherence to chronic diseases [72]. However, the entire medication use process which extends across the borders of several different organizations, had not been evaluated to ensure effective, appropriate, and cost-effective pharmacotherapy. The RPAP provided an opportunity for this comprehensive review and the identification of various development measures (Table s2 in Additional File 5).

Most of the medicines are used in outpatient care, so the measures of the Action Plan are largely focused there and are especially aimed at optimizing pharmacotherapy for chronic diseases and self-managing at home. However, the Action Plan’s goals were unrelated to the operational environment. The evolution of pharmaceutical services within inpatient care has been intensive after the launch of the RPAP. Traditionally, hospital pharmacy services have focused primarily on logistical functions, with only limited integration into clinical practices. The development of the information flow, task definitions, and responsibilities in the medication use process, as well as to defining consistent operating models for medication optimization and management have progressed quickly in inpatient care [73, 74]. The number of ward and clinical pharmacy staff has remarkably increased between 2017 and 2022 in Finland, and services have extended widely, focusing on system-based medication safety work and the development of comprehensive medication management [74]. However, there is a lot of regional variation in development and the goals of the Action Plan are still relevant. Especially in outpatient care, pharmaceutical services except for dispensing, counseling, and automated dose dispensing, are not well integrated into daily clinical practice [75]. The community pharmacies are willing to develop their services improving medication safety and supporting rational pharmacotherapy [76], but non-formed legislation and incentives have hampered the progression. The challenges of the legislation and delays in achieving the goals set in the Action Plan are partially explained by the national social and health services reform, the implementation of which has finally created the conditions for the beginning of legislation development of the pharmaceutical system.

Based on the results of this study, the ultimate goal of developing the pharmaceutical system should be to improve its integration into the social and health services system. In the Action Plan prioritized development activities can be promoted by renewing management, funding, and information sharing in the pharmaceutical, social and health services. During the implementation period of RPAP, the further planning for developing pharmacotherapy data and information management has progressed systematically at national level. MSAH has recently published the enterprise architecture of pharmacotherapy [48] and plans for the development of a centralized national information management services for pharmacotherapy [77] and medicinal data repository [78]. Community pharmacy system’s reform needs (e.g., tasks and operations) have been investigated in detail, also from the general public approach [79]. In the current legislative framework, where community pharmacies are regulated as a separate part of the social and health service system, it is not possible to develop the exchange of patient information, and the tasks of different professionals and organizations in the medication use process agilely as in inpatient care. In addition, the vision has matured that the national level decision-making processes to guide the use of medicines must be developed [80, 81], and the data and information about pharmaceuticals and pharmacotherapy which accumulates in different registers must be utilized better than at present [81, 82].

The Action Plan also highlighted the need for change in governance and funding, which require a new way of thinking to create incentives for various actors in the medication use process to promote rational pharmacotherapy. For example, community pharmacies currently make a profit primarily from the sale of medicines and the definition of profit margins rather than from services that optimize the use of medicines and monitor their effectiveness. The Action Plan set several goals for the regional well-being service counties for managing the medication use process, the governance of the services, and creating conditions for interprofessional and people-centered collaboration. The success of the several goals set for the well-being service counties may require the expansion of their operational mandate to the entire medication use process. Based on the restructuring of the social and health services, the well-being services counties should guide production more strongly than municipalities did before and pay special attention to those who use many services and may also use expensive medicines or several different medicines for their ailments [58]. However, the well-being services counties currently do not have a mandate, e.g., to guide or oblige community pharmacies to develop their services in the medication use process. To make pharmacotherapy more rational, the state and well-being services counties must succeed in integrating the region's community pharmacies into the service chains and by this enable better utilization of available knowledge and resources to medication use optimization and management. The well-being services counties play a central role in many prioritized actions in the Action Plan to promote rational pharmacotherapy. Therefore, they will be a significant player in medicines policy in the future.

The parallel development of the European-level pharmaceutical system increases the challenge of national development. The European Commission has recently published the proposal for the major EU pharmaceutical legislation reform [83]. The proposal complements the key previous changes and initiatives [63, 64]. The goal is to make medicines more equally available, accessible, and affordable in the EU region, boost competitiveness, fight against antimicrobial resistance, and give rules to digital transformation. These phenomena and challenges have partly been identified at the national level during the development of the preceding NMP 2020 [20] and RPAP [50]. Recent global, EU and national level policymaking are more strongly interlinked than before, where e.g., environmental consciousness as a sixth dimension of rational pharmacotherapy is emphasized as a new theme [62, 66, 84, 85].

The strength and uniqueness of the RPAP is the utilization of research in identifying the development needs. Research has not previously been utilized in preparing of medicines policy on the same scale in Finland, although the researchers were also involved in the preparation of the preceding NMP [20]. The importance of evidence-informed decision-making in implementing RPAP is reflected in the long-term research strategy and establishment of a research network to support the implementation of the RPAP [86, 87]. The utilization of academic research could also have influenced the fact that the coverage of the RPAP was comparable to other international NMPs published in other countries [17, 18, 88]. That reflects similar pharmacotherapy challenges in the health systems globally.

The RPAP was the crystallization of NMP thinking as part of the broader social and health policy during one term of government [25]. Currently, there is no updated comprehensive NMP in Finland and the progress of the implementation of the RPAP has also yet to be evaluated. However, to ensure the long-term implementation of the RPAP, a commitment across political party lines has been sought. The officials of the MSAH have developed frameworks for the pharmaceutical system development, which the previous (2019–2022) and current (2023–2027) governments have included in their programs [85, 89]. This commitment aims to ensure continuity and mutual support for long-term and predictable development, transcending different government periods. However, the key question is whether the subsequent governments will commit to a balanced policy that considers the different dimensions of rational pharmacotherapy supporting public health and national health policy goals. It would be appropriate to reform the structures of the pharmaceutical system in a controlled manner and create systemic conditions for implementing rational pharmacotherapy in Finland.

The study results are based on several publicly available documents published by the authorities (Table s1 in Additional File 1) and are consistent with the theoretical framework of integrated care [54]. Only one researcher was responsible for the analyses which is a limitation of this study. However, the interpretations have been validated by authors who have been strongly involved in the RPAP development work and have long experience in national development and working at the interface of politics from different approaches. Each author's point of view in the RPAP development process has been different, enabling the adoption of different perspectives and views during different phases of the analysis, thus strengthening the consensus. However, as a typical limitation to document analysis [57], the documents selected for the analysis and the documents used to verify interpretations have affected the results, i.e., the accuracy and comprehensiveness of the observations made. For example, the development needs to be related to pharmaceutical innovation activities remained few in the results. On the other hand, the pharmaceutical innovation theme was paid less attention due to the more urgent needs to evaluate the effects of the ongoing social and health services reform. The research materials consisted of the central available materials describing the process and the final results of the RPAP. The expertise of authors with the subject of the study compensates for the limitations of document analysis. With the help of the author group, consisting of civil servants and academics, and theories that guided the analysis, it has been possible to strengthen the reliability of the results. The results represent a national case study and are, therefore, not transferable as such to other countries. As presented in this study, the national descriptions of medicines policy and system development holds potential to be useful in several other countries. The results provide an opportunity for cross-country benchmarking and learning.

The major ongoing changes in the domestic and international operational environment affecting the whole pharmaceutical system in Finland require further research. National research in the key areas of rational pharmacotherapy covering structures, processes, and outcomes should be continued, as well as monitoring the impact of the policy measures taken [86]. In addition, the impact of the RPAP on stakeholders’ and patients’ experiences should be investigated. Further research is needed to investigate the prerequisites for integrating community pharmacy services into regional service chains and creating incentives. Internationally, further research is needed on the effectiveness of NMP guidance, and the indicators defined for implementing the NMP.

## Conclusions

Through intensive stakeholder participation, the bottom-up approach created a national vision and principles of rational pharmacotherapy and a strong commitment to implementing the goals and measures. The concern lies in ensuring the continuity of the Action Plan implementation and achieving a balanced long-term development aligned with the integrated national social and health system. The development of the pharmaceutical system has several national and EU-level dependencies requiring long-term political commitment. While the Action Plan differs from the national medicines policy it forms a good basis for long-term development covering important parts of medicine policy at the micro, meso and macro levels of the service system.

### Supplementary Information

Supplementary Material 1. 

Supplementary Material 2. 

Supplementary Material 3. 

Supplementary Material 4. 

Supplementary Material 5. 

Supplementary Material 6. 

Supplementary Material 7. 

## Data Availability

Publicly available reports from open sources: https://julkaisut.valtioneuvosto.fi/ and https://stm.fi/rationaalinen-laakehoito/julkaisut were used. Meeting memos of the RPAP Steering Group were made available through a formal information request to the Ministry of Social Affairs and Health (VN/22279/2022). The data that support the findings of this study are available from the corresponding author upon reasonable request.

## Abbreviations

EMA: European Medicines Agency
EU: European Union
Fimea: Finnish Medicines Agency
GDP: Gross Domestic Product
HTA: Health Technology Assessment
MSAH: Ministry of Social Affairs and Health
NMP: National Medicine Policy
OTC: Over the Counter (medicine)
RPAP: Rational Pharmacotherapy Action Plan
RWD: Real-World Data
WG: Working Group
WHO: World Health Organization
