# Supplementary material for: Drawing up the public national Rational Pharmacotherapy Action Plan as part of social and health services reform in Finland: a bottom-up approach involving stakeholders
Source: BMC Health Serv Res. 2024 May 16;24:631. doi: 10.1186/s12913-024-11068-y (PMC11097518; doi:10.1186/s12913-024-11068-y)
Supplement: Supplementary file 1 — Supplementary Material 1. [file 12913_2024_11068_MOESM1_ESM.docx]

Additional File 1 - Material used in content analysis and to strengthen its interpretations

**Table s1** Material that was qualitatively content analyzed and documents that were utilized in interpretation. MSAH = Ministry of Social Affairs and Health, NPP = New pharmaceutical product, RPSP = Rational Pharmacotherapy Action Plan, WG = Working Group.

| **No.** | **Authors** | **Title** | **Method of the report** | **Content or Outcomes** | **Availability** |
| --- | --- | --- | --- | --- | --- |
| ***Material of the content analysis*** | | | | | |
| 1. | Hämeen-Anttila K, Närhi U, Tahvanainen H. | Final Report, Rational Pharmacotherapy Action Plan | Synthesis of WG reports and stakeholder cooperation | ▪ Identifying the key themes relating to the use of medicines  ▪ Outlining the objectives for promoting rational pharmacotherapy for national implementation, services organizers (commissioning) and providers, healthcare and social welfare professionals, and medicine users.  ▪ Defining the concept of rational pharmacotherapy | Reports and Memorandums of the Ministry of Social Affairs and Health, English version (2018:19), Available at: <http://urn.fi/URN:ISBN:978-952-00-3930-1> |
| 2. | Steering Group of the RPAP. Ministry of Social Affairs and Health (2016-2018) | Steering Group memos I-XVI | - | ▪ Steering Group meeting I on the 1^st^ of Feb 2016  ▪ Steering Group meeting II on the 6^th^ of April 2016  ▪ Steering Group meeting III on the 9^th^ of May 2016  ▪ Steering Group meeting IV on the 13^th^ of June 2016  ▪ Steering Group meeting V on the 22^nd^ of August 2016  ▪ Steering Group meeting VI on the 26^th^ of Sep 2016  ▪ Steering Group meeting VII on the 24^th^ of October 2016  ▪ Steering Group meeting VIII on the 28^th^ of Nov 2016  ▪ Steering Group meeting IX on the 7^th^ of Dec 2016  ▪ Steering Group meeting X on the 23rd of January 2017  ▪ Steering Group meeting XI on the 27^th^ of Feb 2017  ▪ Steering Group meeting XII on the 29^th^ of March 2017  ▪ Steering Group meeting XIII on the 17^th^ of May 2017  ▪ Steering Group meeting XIV on the 19^th^ of June 2017  ▪ Steering Group meeting XV on the 12^th^ of October 2017  ▪ Steering Group meeting XVI on the 8^th^ of Dec 2017  ▪ RPAP publishing seminar on the 20^th^ of March 2018 | The Steering Group meeting memos were made available through an information request to the MSAH |
| ***WG Reports of the RPAP were used to verify the interpretations in this study*** | | | | | |
| 3. | WG1, Ruokoniemi P. (editor) | Report of the Rational Prescribing, Dispensing and Use of Medicines | Literature review, a synthesis of expert opinions | ▪ Identifying areas for development: control of cost growth, up-to-date availability of patient-specific information, management of the entirety of medication regimen, guiding prescribing, increasing equality as part of the implementation of social and health care reform  ▪ In the future, the prescribing, distributing, and using medicines will be based on rational pharmacotherapy goals set nationally and supplemented regionally. | Reports and Memorandums of the Ministry of Social Affairs and Health (2018:12), Available in Finnish with English abstract at: <http://urn.fi/URN:ISBN:978-952-00-3912-7> |
| 4. | WG2, von Bonsdorff-Nikander A, Närhi U, Särkkä E, Tahvanainen H. (editors) | Report of the Pharmaceutical services in the operating environment of health and social services | A synthesis of expert opinions | ▪ Identifying areas for development: integration, service production legislation and operating models, national and regional coordination, and steering, ensure equal access to medicines  ▪ Development of a pharmaceutical system to enhance rational pharmacotherapy: objectives of a sufficient amount of community pharmacies and converging operating models at different levels of service system, improvement of the medication safety, establishment of the national medicine information center  ▪ Assessment of the effects of EU legislation regarding the national service system and the reforms of the national pharmaceutical system | Reports and Memorandums of the Ministry of Social Affairs and Health (2018:6), Available in Finnish with English abstract at: <http://urn.fi/URN:ISBN:978-952-00-3903-5> |
| 5. | WG3, Rannanheimo P. (editor) | Report on How should the assessment of the therapeutic and economic value of medicines be organized in Finland's health and social services? | A synthesis of expert opinions | ▪ The report focuses on medicines used mainly in public healthcare hospitals  ▪ The assessment of pharmacotherapies should better support decision-making, procurement procedures, and determining the price of the medicinal products  ▪ Additional evidence on the consumption, effects, targeting of use, treatment process, and costs of the evaluated medicines should be collected regularly from the healthcare registers and databases  ▪ Cooperation with clinical experts should be increased | Reports and Memorandums of the Ministry of Social Affairs and Health (2017:31), Available in Finnish with English abstract at: <https://julkaisut.valtioneuvosto.fi/handle/10024/80775> |
| 6. | WG4 Airaksinen M, Saastamoinen L, Hämeen-Anttila K. | Report on Research for Evidence-Informed Decision-Making: Research Strategy for Rational Pharmacotherapy | Literature review, a synthesis of expert opinions | The objective, by 2022: ▪ Research on and development of rational pharmacotherapy is a part of the social and health services system  ▪ Research is utilized diversely in informing decision-making in the social and health services system and medicines policy  ▪ Research and allocation of resources to the research areas presented in the research strategy are strong  ▪ The research areas: 1) structures and operating preconditions, 2) medication process, 3) medicines use and effectiveness and economy of pharmacotherapy | Reports and Memorandums of the Ministry of Social Affairs and Health (2018:25), Available at: <http://urn.fi/URN:ISBN:978-952-00-3940-0> |
| 7. | WG5 Tahvanainen H. (editor) | Report on National Pharmaceutical Development Centre | A synthesis of expert opinions and preliminary report (Palva E. 2017) | ▪ A national drug development center is proposed to be established, which would equally serve all researchers in, for example, universities, research institutes, and hospitals  ▪ The aim is to enhance drug development projects based on academic research to the clinical stage of the drug development cycle and to create new growth companies | Reports and Memorandums of the Ministry of Social Affairs and Health (2018:5), Available in Finnish with English abstract at: <http://urn.fi/URN:ISBN:978-952-00-3902-8> |
| 8. | WG6 Tahvanainen H. (editor) | Report on the Development of data management to support rational pharmacotherapy | A synthesis of expert opinions | ▪ The focus of the report is to recognize information management development needs from the perspectives of  1) Medication user: a national up-to-date medication list, digital treatment path, the opportunities for citizens and healthcare professionals to identify problems with pharmacotherapy  2) Service organizer: be able to produce key figures for pharmacotherapy and pharmaceutical systems, i.e., indicator information for the needs of guidance and control and for evaluating the effectiveness of NPP  ▪ The report describes the identified areas of development regarding the functionality of the various systems | Reports and Memorandums of the Ministry of Social Affairs and Health (2018:5), Available in Finnish with English abstract at: <http://urn.fi/URN:ISBN:978-952-00-3911-0> |
| ***Expert reports ordered by MSAH were used to verify the interpretations in this study*** | | | | | |
| 9. | Torniainen K. | Preliminary report: Hospital Pharmacy in future service systems structures | Expert report based on the surveys addressed to hospital pharmacies | The outcomes of the report:  ▪ Alongside the structural reforms, some of the current licenses of hospital pharmacies and medical centers could be abolished  ▪ One license should enable the maintenance of several pharmaceutical service units, and the investments made should be considered in the location of the offices  ▪ Stronger coordination is recommended so that cooperative areas would be responsible for the regional coordination of pharmaceutical care and logistics in the public sector  ▪ The coordination would mean responsibility for strategic tasks related to procurement, medication and patient safety, and preparedness, as well as coordination in the development of the information systems  ▪ Regarding pharmaceutical manufacturing in hospitals, a separate survey should be carried out | Reports and Memorandums of the Ministry of Social Affairs and Health (2018:13), Available only in Finnish at: <http://urn.fi/URN:ISBN:978-952-00-3913-4> |
| 10. | Palva E. | National Center for the Drug Development, Preliminary report | Expert report based on the interviews addressed to different stakeholders | ▪ The report aimed to find out 1) Why should a drug development center be established? 2) What would the concept of a drug development center be like? 3) What would the task of the center be? 4) What could be the financial basis of the center in the initial stages of operation and later? 5) What would be the size of the center and its personnel? 6) What would the organization be like, and who would manage the center? 7) What should be clarified in the future?  ▪ As a result, a national drug development center is proposed to be established | Reports and Memorandums of the Ministry of Social Affairs and Health (2017:15), Available only in Finnish at: <http://urn.fi/URN:ISBN:978-952-00-3865-6> |
| 11. | Kvarnström K. | Study on the activities of the Swedish pharmacotherapy working groups and suggestions to those who have the organizing role after the national social and health service reform | Content analysis of the collected material from the websites of Swedish authorities and working groups, scientific articles | ▪ Finland should consider the establishment of pharmaceutical working groups like those in Sweden  ▪ The operational model of the pharmaceutical working groups could utilize the previous experiences on workshop and network methods in Finland as well as experiences from Sweden  ▪ The national pharmacotherapy working group should coordinate the activities of the work in the cooperation areas  ▪ Respectively, pharmacotherapy working groups of cooperation areas should coordinate the rational pharmacotherapy in the well-being services counties and the local medication process through multi-professional networks, also having representation from community pharmacies | Ministry of the Social Affairs and Health. Available only in Finnish at: <https://stm.fi/documents/1271139/7229376/Selvitys+Ruotsin+l%C3%A4%C3%A4kety%C3%B6ryhmien+toiminnasta_FINAL.pdf/66fb0ac4-790e-402b-9610-0e29059df59f/Selvitys+Ruotsin+l%C3%A4%C3%A4kety%C3%B6ryhmien+toiminnasta_FINAL.pdf?t=1520853059000> |
| 12. | Kiviluoto K. | International comparison (Sweden, Denmark, Scotland, Finland) of the means to improve rational pharmacotherapy | Content analysis of the literature and web pages | ▪ Various means to improve rational pharmacotherapy have been introduced: medication prescription monitoring, national and regional formularies, budget targets, academic detailing, recommendations, national electronic health records, generic substitution, INN-prescribing,  ▪ Sweden and Great Britain use financial incentives to guide prescribing, but the evidence is contradictory  ▪ To improve medication safety, the flow of information and learning from mistakes that have occurred are important  ▪ Reliable and independent medicines information is essential to ensure rational pharmacotherapy | Ministry of the Social Affairs and Health. Available only in Finnish at: <https://stm.fi/documents/1271139/7229376/RATIONAALISEN+L%C3%84%C3%84KEHOIDON+OHJAAMISEN+KOKEMUKSIA.pdf/e90a2437-af71-4a2e-9677-5daf53f288a8/RATIONAALISEN+L%C3%84%C3%84KEHOIDON+OHJAAMISEN+KOKEMUKSIA.pdf> |
| 13. | Kortejärvi H, Kunnamo I. | Suggestions for the pharmacotherapy management and optimization | Expert report | ▪ The national medication list and the comprehensive treatment plan, including a pharmacotherapy plan, are means to manage the prescribing, implementation, and monitoring of the effects of pharmacotherapy  ▪ Decision support is a useful tool for rational prescribing, identifying those with medication-related problems, and evaluating of pharmacotherapy  ▪ Customers and staff working in social and healthcare units receive counseling from hospital and community pharmacies  ▪ Social and health service providers use the expertise of pharmacists and organizational pharmacotherapy plans, self-monitoring, and indicators as tools for guiding and developing operations to improve rational pharmacotherapy  ▪ Development of clinical pharmacy expertise and multi-professional cooperation in medication review are practical means for managing medication regimens on personal level  ▪ A national center that coordinates medication safety and rational pharmacotherapy should be established.  ▪ Medication safety coordinators should work in each well-being services county.  ▪ National criteria and indicators are needed to rational pharmacotherapy implementation and promote medication safety | Ministry of the Social Affairs and Health. Available only in Finnish at: <https://stm.fi/documents/1271139/3206721/Ehdotuksia+l%C3%A4%C3%A4kehoidon+kokonaisuuden+hallintaan+ja+optimointiin_260319_B.pdf/964331f6-40e4-c108-4126-b02655b59fc5/Ehdotuksia+l%C3%A4%C3%A4kehoidon+kokonaisuuden+hallintaan+ja+optimointiin_260319_B.pdf?t=1553607327000> |

*MSAH also ordered an investigation report on the effects of EU legislation on the national pharmaceutical system, but this report has not been used in the analysis.*
