# Supplementary figures and images for: Drawing up the public national Rational Pharmacotherapy Action Plan as part of social and health services reform in Finland: a bottom-up approach involving stakeholders
Source: BMC Health Serv Res. 2024 May 16;24:631. doi: 10.1186/s12913-024-11068-y (PMC11097518; doi:10.1186/s12913-024-11068-y)

Additional File 2 – Visualization of the content of the Rational Pharmacotherapy Action Plan (50).


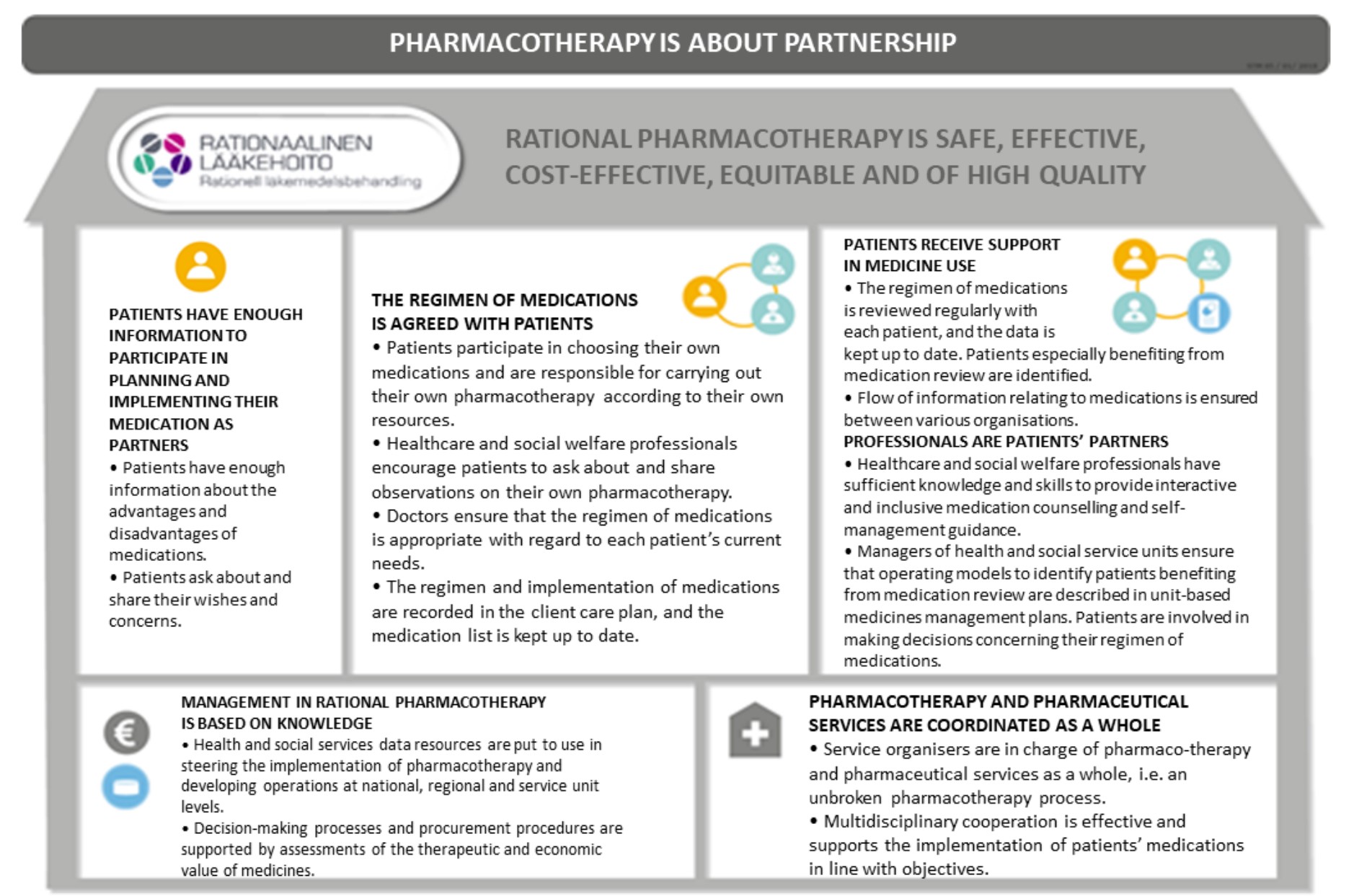

Supplement: Supplementary file 2 — Supplementary Material 2. [file 12913_2024_11068_MOESM2_ESM.docx]
