# Supplementary material for: Drawing up the public national Rational Pharmacotherapy Action Plan as part of social and health services reform in Finland: a bottom-up approach involving stakeholders
Source: BMC Health Serv Res. 2024 May 16;24:631. doi: 10.1186/s12913-024-11068-y (PMC11097518; doi:10.1186/s12913-024-11068-y)
Supplement: Supplementary file 4 — Supplementary Material 4. [file 12913_2024_11068_MOESM4_ESM.docx]

Additional File 4 – Checklist of rational pharmacotherapy elements for healthcare professionals to discuss with medicine users.


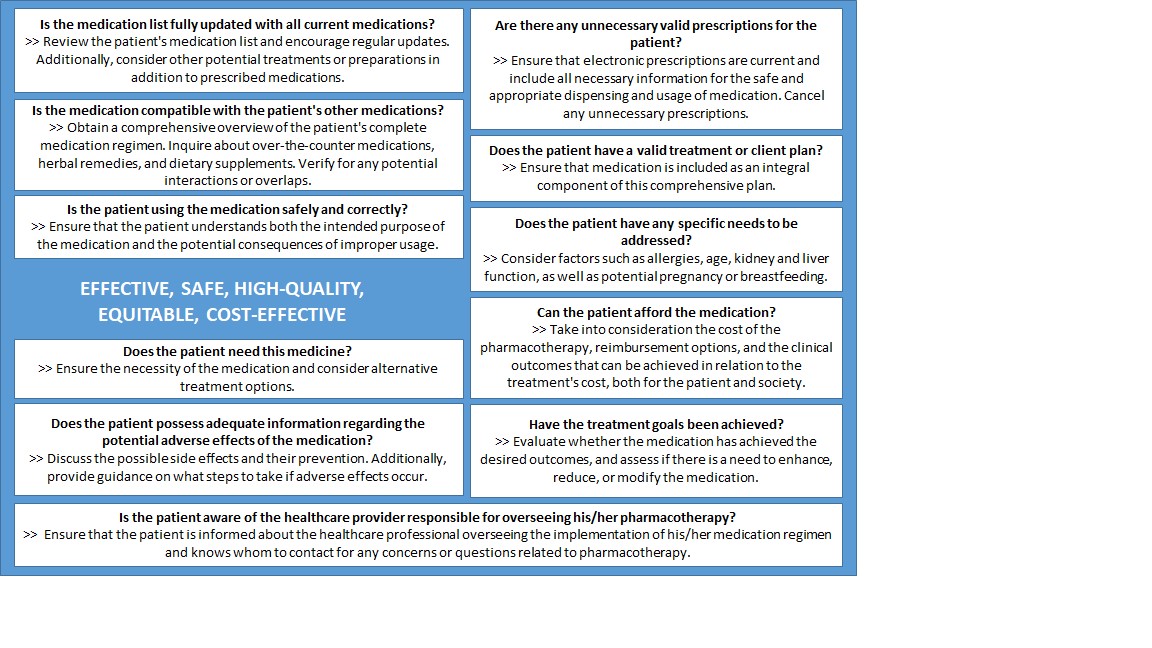


Translated from the source: WG1 Report of the Rational Prescribing, Dispensing and Use of Medicines. Reports and Memorandums of the Ministry of Social Affairs and Health (2018:12). Available in Finnish with English abstract at: <http://urn.fi/URN:ISBN:978-952-00-3912-7>
