# Supplementary material for: Drawing up the public national Rational Pharmacotherapy Action Plan as part of social and health services reform in Finland: a bottom-up approach involving stakeholders
Source: BMC Health Serv Res. 2024 May 16;24:631. doi: 10.1186/s12913-024-11068-y (PMC11097518; doi:10.1186/s12913-024-11068-y)
Supplement: Supplementary file 5 — Supplementary Material 5. [file 12913_2024_11068_MOESM5_ESM.docx]

Additional File 5 – The necessary long-term development to promote rational pharmacotherapy

**Table s2** Prioritized actions of the Action Plan and the means of functional integration required to achieve the normative vision and principles of rational pharmacotherapy (41, 45). F = Functional integration through Funding, HTA = Health Technology Assessment, I = Functional integration through Information, IT = Information Technology, M = Functional integration through Management, NPP = New Pharmaceutical Product, RWD = Real-World Data.

|  | | **The principle of people-centeredness** | **The principle of partnership** | **The principle of evidence-informed policy** |
| --- | --- | --- | --- | --- |
| **1^st^ Normative long-term development vision: Comprehensive medication management based on people-centered interventions and interprofessional collaboration** | | | | |
| **Micro-level prioritized actions** | **Medicine users*** | ▪ Have a clear understanding of their medication regimen and why and how medicines are used. (M)  ▪Take responsibility for the pharmacotherapy according to their own resources. Adopt the medication regimen as agreed and monitor its effects. (M) | ▪ Actively talk about their wishes and concerns related to pharmacotherapy. (M) | ▪ Can easily find and utilize reliable medicine information when visiting social and health service units or pharmacies. (I)  ▪ An up-to-date medication list is a tool that helps a medicine user implement pharmacotherapy and monitor its effects. (I)  ▪ Can record their own health, well-being and medication information and share those with social and healthcare professionals. (I) |
|  | **Social and  healthcare professionals** | ▪ The views of the medication user guide the choices made by different health and social care professionals. (M)  ▪ Prescribers are responsible for the overall management of medication regimens and involve medication users to agree on the treatment selection, goal setting, monitoring, and adopting self-care. (M)  ▪ When prescribing and choosing a pharmaceutical product, special attention is paid to the costs of the medication to the user and society, as well as the clinical effects that can be achieved with the price of the treatment. (M, F) | ▪ Professionals meet medication users as a partner and encourage them to ask about and share their observations about their medication and to make choices that promote rational pharmacotherapy and continuity of treatment. (M)  ▪ Professionals know how to utilize each other’s expertise and form a multidisciplinary team whose cooperation ensures the appropriateness of pharmacotherapy. (M) | ▪ Professionals can fluently use customer and patient information systems and decision support applications to ensure the appropriateness of pharmacotherapy and keep the medication list up to date. (M, I)  ▪ The IT systems guide recording and provide the necessary information about the medication user, treatment recommendations, pharmaceutical products, and treatment plan to guide the work of different professionals in the distinct phases of the medication use process: prescribing, dispensing, counseling, implementing, and monitoring. (M, I)  ▪ A national feedback system for prescription practices supports the development of the competence of prescribers and promotes appropriate decisions. (M, I) |
| **2^nd^ Normative long-term development vision: Management of medication use process and governance of the pharmaceutical services as a unified entity** | | | | |
| **Meso-level prioritized actions** | **Service providers and service units** | ▪ The management of service production is based on the customer-oriented approach in the implementation and development of the service. (M, F)  ▪ The treatment plan and, as part of it, the pharmacotherapy plan is up-to-date, especially for those who use many services or medicines or have expensive therapies. (M, I)  ▪ In addition to dispensing and counseling practices, outpatient care pharmacies support medication management with clinical pharmacy services. (M, F) | ▪ The entire medication use process is defined, controlled, and monitored to promote rational pharmacotherapy and to control the preventable risks of pharmacotherapy. (M, I)  ▪ Management emphasizes the multidisciplinary cooperation that crosses organizational boundaries, is based on operational models that promote rational prescribing, dispensing, and use of medicines, the appropriate division of work between different professionals, and ensures the continuity of treatment. (M, F) | ▪ Information systems that support work, and alarms and reminders that guide workflow for different professionals. (I)  ▪ The information flow is fluent, and exchanging necessary patient data and messages between service units and community pharmacies is possible. (I)  ▪ To support management, guidance, and development, compiled information is available on various themes, such as prescription practices, adherence to medication, medication errors, audit findings, current treatment indicators, medication cost, and continuity of treatment. (M, I) |
|  | **Well-being service counties** | ▪ Monitor, evaluate, guide, and develop the implementation of rational pharmacotherapy in the service entities and chains based on the organizing responsibilities, and also consider the operations of community pharmacies. (M, F, I)  ▪ Organizes and allocates services that support medication regimen management for people who use many services, medicines, or expensive medicines. (M, F, I)  ▪ Ensures the medication use processes are uninterrupted to achieve the welfare outcomes of the medication users in all service entities and chains. (M)  ▪ Ensures regionally and, if necessary, nationally interoperable information systems that support the management of personal pharmacotherapy and preventable medication risks, as well as the uniform recording of different professionals. Special attention is paid to the usability of the systems. (M, I) | ▪ Responsible for implementing the principles of rational prescribing and use of medicines. Create the necessary incentives for service providers to promote rational pharmacotherapy. (M, F)  ▪ Public pharmaceutical services (procurement, storage, dispensing, manufacturing, clinical pharmacy, and clinical trial services) are organized efficiently and productively, considering the differences between different regions, the cooperation of hospital pharmacies and community pharmacies, and the requirements of emergency and preparedness operations. (M, F, I)  ▪ The availability of community pharmacy services in each well-being services county is sufficient, and the counties utilize resources of community pharmacies on a contract basis to promote rational pharmacotherapy. (M, F, I) | ▪ The population-based management and decision-making of social and health services are guided by regional and national comparison information collected from service production, including pharmaceutical services, as well as expert assessment that describes the use and costs of medicines, effects, continuity, equality, and the safety of treatment. (M, I)  ▪ Abilities for utilizing information and promoting rational pharmacotherapy 1) at distinct phases of the life cycle of the pharmaceutical product, 2) between the different treatment options, and 3) in different patient groups. (M, I, F)  ▪ The NPP or indication extension introduction procedures are harmonized within the well-being services counties. (M)  ▪ Research and development of rational pharmacotherapy supports the development of clinical practices, processes, and service entities. (M) |
| **3^rd^ Normative long-term development vision: Evidence-informed steering and decision-making on pharmacotherapy and pharmaceutical services** | | | | |
| **Macro-level prioritized actions** | **National authorities** | ▪ The guidance that is part of the tasks of various authorities is based on the national concept of rational pharmacotherapy, treatment recommendations, and considers the perspectives of both the individuals and society. (M)  ▪ National descriptions of the operating models of medication management interventions and the criteria for identifying people who benefit from them are in use. (M) | ▪ Nationally, the coordination and division of work in some areas of responsibility are clarified, such as the development of medicine information and medicine information services, guiding the introduction of NPP and the withdrawal of irrational ones, developing and guiding medication safety, responsibility for securing the availability of essential medicines and patient-specific manufacturing in all conditions, and international cooperation in different themes of rational pharmacotherapy. (M)  ▪ Medication safety knowledge and understanding of the principles of rational pharmacotherapy are part of the professional basic, continuing, and supplementary training. (M)  ▪ National measures improve the development and commercialization possibilities of pharmaceutical innovations. (M, F) | ▪ The information in the various national data repositories should be developed to be comprehensive, high-quality, usable, and easy to compile for different purposes. (I)  ▪ Nationally produced statistical and indicator data support rational pharmacotherapy at the micro, meso and macro levels. (M, I)  ▪ Structured and planned routine RWD-based additional data collection and timely HTA support the management of the lifecycle costs of an individual pharmaceutical product in evidence-based decision-making. (M, I, F)  ▪ The information recorded in the distinct phases of the medication process by professionals and medicine users can be utilized for secondary use, such as research, development, education and innovation activities, management, and decision-making within the approved regulation framework. (M, I) |
|  | **European Union** | ▪ Evolving EU regulation and interoperable national operational models have a role in safeguarding medication users’ rights and facilitating the implementation of rational pharmacotherapy, benefiting both individuals and society. (M, F, I) | ▪ The harmonization and movement of health data and medicinal product information across borders aims to facilitate the functioning European pharmaceutical market, where Member States have the autonomy to determine how to allocate resources for medicines use, as well as principles to monitor, evaluate, and guide the implementation of rational pharmacotherapy. (M, F, I) | ▪ The development of the European data infrastructure in cooperation with the various national and EU authorities and agencies facilitates a national vision for utilizing all available information in decision-making.  (M, I) |

* *Medicine users or their relatives depending on their functional and cognitive ability*
