# Supplementary material for: Drawing up the public national Rational Pharmacotherapy Action Plan as part of social and health services reform in Finland: a bottom-up approach involving stakeholders
Source: BMC Health Serv Res. 2024 May 16;24:631. doi: 10.1186/s12913-024-11068-y (PMC11097518; doi:10.1186/s12913-024-11068-y)
Supplement: Supplementary file 6 — Supplementary Material 6. [file 12913_2024_11068_MOESM6_ESM.docx]

Additional File 6 – An example of material classification

**Table s3** Example of the coding tree for the deductive content analysis

| **The main classification** | **Parent classification** | **Sub classification** | **Reduced expression** | **Excerpts from the material** |
| --- | --- | --- | --- | --- |
| Prioritized actions to achieve principles of rational pharmacotherapy | Meso-level of the system | Well-being service county | Monitor, evaluate, guide, and develop the implementation of rational pharmacotherapy in the service entities and chains based on the organizing responsibilities, and also consider the operations of community pharmacies. | (Final Report, see Additional file 1): “In the future social and health service structures, service organizers are responsible for ensuring that citizens receive the social and healthcare services they need. Pharmacotherapy is a part this whole: the service organizers are therefore responsible for pharmacotherapy and pharmaceutical services as a whole entity, the coordination of medication safety and medicines information, the safety monitoring and evaluation of the use of medicines and the functionality of the pharmaceutical services.”  “The service organizer is partly responsible for ensuring that the availability of community pharmacy services is sufficient. Community pharmacies operate as part of the social and health service and treatment path”.  “National guidelines, evidence-based treatment recommendations and other jointly agreed goals are used as the basis for guidance.” |
|  |  |  |  | (WG 1 report, see Additional File 1): “Prescribing, supply and use of medicines are based to national and regional goals… National goals are specified regionally, and goals achievement is monitored centrally. This ensures the equality of medicine users in Finland.” |
|  |  |  |  | (WG 2 report, see Additional File 1): “The responsibility for organizing services includes the entirety of pharmaceutical services (hospital pharmacies and community pharmacies). This is also covering the availability of clinical pharmacy services that support the management of medication use process and patient-specific optimization of the pharmacotherapy, and the need of those services in different operating environments. |
| Prioritized actions to achieve principles of rational pharmacotherapy | Macro -level of the system | National Authority | National descriptions of the operating models of medication management interventions and the criteria for identifying people who benefit from them are in use. | (Final Report, see Additional file 1): “National operational models for medication reconciliation, identifying medicine users who benefit from medication review, and targeting medication review intervention have been drawn up.” |
|  |  |  |  | (WG 1 report, see Additional File 1): “Regarding the implementation of medication review measures and evaluations, a national, multi-professional consensus and operating models should be created in the future, which would clarify how the expertise and resources of different professionals are obtained in practice, best for beneficial use, i.e. to support patients in managing the entirety of medication regimen. At the same time, it is necessary to think about the implementation of functional, even cross-organizational, multi-professional cooperation in and between different operating environments, for example between community pharmacies, public health centers, private medical clinics, hospital pharmacies and public hospitals”. |
|  |  |  |  | (Kortejärvi and Kunnamo expert report, see Additional File 1): “In the future, the risks and needs for changes in the medication regimen of long-term and multi-morbid patients screening tools, which help in identification from the population, those persons whose medication is associated with risks and who can benefit from a medication review”. |
