# Supplementary material for: Drawing up the public national Rational Pharmacotherapy Action Plan as part of social and health services reform in Finland: a bottom-up approach involving stakeholders
Source: BMC Health Serv Res. 2024 May 16;24:631. doi: 10.1186/s12913-024-11068-y (PMC11097518; doi:10.1186/s12913-024-11068-y)
Supplement: Supplementary file 7 — Supplementary Material 7. [file 12913_2024_11068_MOESM7_ESM.docx]

Additional File 7 - Consolidated criteria for reporting qualitative studies (COREQ): 32-item checklist

| **No. Item** | **Guide questions/description Reported** | **Reported on page number/paragraph and section** |
| --- | --- | --- |
| **Domain 1: Research team and reflexivity** | | |
| *Personal Characteristics* | | |
| 1. Interviewer/facilitator | Which author/s conducted the interview or focus group? | N/A |
| 2. Credentials | What were the researcher’s credentials? E.g. PhD, MD | Page 2-3/Authors |
| 3. Occupation | What was their occupation at the time of the study? | Page 2-3/Authors |
| 4. Gender | Was the researcher male or female? | Page 42/Authors’ information |
| 5. Experience and training | What experience or training did the researcher have? | Page 2-3/Authors |
| *Relationship with participants* | | |
| 6. Relationship established | Was a relationship established prior to study commencement? | page 14/ A qualitative synthesis of data/Paragraph 3 |
| 7. Participant knowledge of the interviewer | What did the participants know about the researcher? e.g. personal goals, reasons for doing the research | N/A |
| 8. Interviewer characteristics | What characteristics were reported about the interviewer/facilitator? e.g. Bias, assumptions, reasons and interests in the research topic | N/A |
| **Domain 2: study design** | | |
| *Theoretical framework* | | |
| 9. Methodological orientation and Theory | What methodological orientation was stated to underpin the study? e.g. grounded theory, discourse analysis, ethnography, phenomenology, content analysis | page 12/ Theoretical framework of this study/Paragraph 1-3 and page 13/Study design and methods/Paragraph 1 |
| *Participant selection* | | |
| 10. Sampling | How were participants selected? e.g. purposive, convenience, consecutive, snowball | N/A |
| 11. Method of approach | How were participants approached? e.g. face-to-face, telephone, mail, email | N/A |
| 12. Sample size | How many participants were in the study? | N/A |
| 13. Non-participation | How many people refused to participate or dropped out? Reasons? | N/A |
| *Setting* | | |
| 14. Setting of data collection | Where was the data collected? e.g. home, clinic, workplace | N/A |
| 15. Presence of non-participants | Was anyone else present besides the participants and researchers? | N/A |
| 16. Description of sample | What are the important characteristics of the sample? e.g. demographic data, date | N/A |
| *Data collection* | | |
| 17. Interview guide | Were questions, prompts, guides provided by the authors? Was it pilot tested? | N/A |
| 18. Repeat interviews | Were repeat interviews carried out? If yes, how many? | N/A |
| 19. Audio/visual recording | Did the research use audio or visual recording to collect the data? | N/A |
| 20. Field notes | Were field notes made during and/or after the interview or focus group? | N/A |
| 21. Duration | What was the duration of the interviews or focus group? | N/A |
| 22. Data saturation | Was data saturation discussed? | N/A |
| 23. Transcripts returned | Were transcripts returned to participants for comment and/or correction? | N/A |
| **Domain 3: analysis and findings** | | |
| *Data analysis* |  |  |
| 24. Number of data coders | How many data coders coded the data? | page 14/ A qualitative synthesis of data/Paragraph 3 |
| 25. Description of the coding tree | Did authors provide a description of the coding tree? | page 13-14/ A qualitative synthesis of data/Paragraph 1-2, page 51-52/Additional File 6/Table s3 |
| 26. Derivation of themes | Were themes identified in advance or derived from the data? | page 11-12/ A qualitative synthesis of data/Paragraph 1-2 |
| 27. Software | What software, if applicable, was used to manage the data? | MS excel |
| 28. Participant checking | Did participants provide feedback on the findings? | N/A |
| *Reporting* |  |  |
| 29. Quotations presented | Were participant quotations presented to illustrate the themes / findings? Was each quotation identified? e.g. participant number | N/A |
| 30. Data and findings consistent | Was there consistency between the data presented and the findings? | page 48-50/Additional File 5/Table s2 |
| 31. Clarity of major themes | Were major themes clearly presented in the findings? | page 19-21/Outcomes and core contents of the Rational Pharmacotherapy Action Plan development process/Paragraph 2-3 |
| 32. Clarity of minor themes | Is there a description of diverse cases or discussion of minor themes? | page 21-23/Outcomes and core contents of the Rational Pharmacotherapy Action Plan development process/Paragraph 3-4 |

The check* list has been applied to the reporting of the results of this qualitative study, which used reports published by authorities and working group memos of Rational Pharmacotherapy Action Plan Steering Group as research material.

*) Tong A, Sainsbury P, Craig J. Consolidated criteria for reporting qualitative research (COREQ): a

32-item checklist for interviews and focus groups. International Journal for Quality in Health Care.

2007. Volume 19, Number 6: pp. 349 – 357
